# The Epiphytic Fern *Elaphoglossum luridum* (Fée) Christ. (Dryopteridaceae) from Central and South America: Morphological and Physiological Responses to Water Stress

**DOI:** 10.1155/2014/817892

**Published:** 2014-10-20

**Authors:** Bruno Degaspari Minardi, Ana Paula Lorenzen Voytena, Marisa Santos, Áurea Maria Randi

**Affiliations:** Department of Botany, Federal University of Santa Catarina, CP 476, 88049-900 Florianópolis, SC, Brazil

## Abstract

*Elaphoglossum luridum* (Fée) Christ. (Dryopteridaceae) is an epiphytic fern of the Atlantic Forest (Brazil). Anatomical and physiological studies were conducted to understand how this plant responds to water stress. The *E. luridum* frond is coriaceus and succulent, presenting trichomes, relatively thick cuticle, and sinuous cell walls in both abaxial and adaxial epidermis. Three treatments were analyzed: control, water deficit, and abscisic acid (ABA). Physiological studies were conducted through analysis of relative water content (RWC), photosynthetic pigments, chlorophyll a fluorescence, and malate content. No changes in RWC were observed among treatments; however, significant decreases in chlorophyll a content and photosynthetic parameters, including optimal irradiance (*I*_opt_) and maximum electron transport rate (ETR_max_), were determined by rapid light curves (RLC). No evidence of crassulacean acid metabolism (CAM) pathway was observed in *E. luridum* in response to either water deficit or exogenous application of ABA. On the other hand, malate content decreased in the *E. luridum* frond after ABA treatment, seeming to downregulate malate metabolism at night, possibly through tricarboxylic acid (TCA) cycle regulation.

## 1. Introduction

Even in humid rainforests, epiphytic plants without direct contact with the soil are exposed to recurrent drought, and xerophytic features have been found and studied from many distinct taxonomic groups [1]. Epiphytes comprise approximately 10% of the vascular flora, being found almost exclusively in tropical forests, which provide up to 25% of species [2, 3]. According to Madison [4], epiphytic plants do not have direct connections with the ground. In an ecological context, epiphytism is an interaction between plants in which the epiphytic plant is dependent on the substrate supplied by the host plant (phorophyte), while obtaining nutrients directly from atmospheric moisture, without emitting haustorium structures [5]. Epiphytism favors the capture of light irradiation, limiting, at the same time, the availability of water for plants [6, 7].

Study of the ecophysiology of epiphytes has received considerable attention, especially for the adaptations that enable their survival on phorophytes with limited water and no root contact with the soil. These studies are mainly concentrated in the Bromeliaceae [8], although the number of epiphytic bromeliads is fewer than half the number of epiphytic ferns [7]. Epiphytism is pronounced among ferns in that about 29% of fern species regularly occur in tree crowns [7]. While about a third of all ferns are classified as epiphytes, only a small proportion of these can be classified as xerophytic, and separating these from mesic species is particularly difficult [7, 9].

Among the wide variety of epiphytic ferns of the Brazilian forests,* Elaphoglossum luridum* (Fée) Christ. (Dryopteridaceae) is particularly notable [10]. It occurs preferentially in humid forests on large trees. Among pantropical genera,* Elaphoglossum* is one of the largest, containing about 600 species, mostly in the Neotropics, and some 85% of these species are epiphytes [7, 11]. The name of the genus derives from the Greek elaphos (deer) + glossa (tongue), so named for the shape of its fronds. According to Maciel and Pietrobom [12],* E*.* luridum* is distinguished from other species of the genus by its leathery bladed epidermis and ribs with black scales, on both surfaces, especially in the basal region. This species occurs in Costa Rica, Panama, the Lesser and Greater Antilles, Trinidad, Guiana, French Guiana, Suriname, Colombia, Ecuador, Peru, and Bolivia. In Brazil, it is found in Amazonas, Pará, Minas Gerais, Rio de Janeiro, São Paulo, Paraná, Santa Catarina, and Rio Grande do Sul [12].

Drought tolerance is gained through adaptations in water uptake, water loss, water storage, and, in many ferns, desiccation tolerance, which can be divided into two major groups: the poikilohydrous (desiccation-tolerant) and the homoiohydrous (relatively desiccation-intolerant) species [7, 9]. Xerophytes are homoiohydrous and can be drought endurers and drought avoiders [7, 9]. They can show productive ephemeral foliage suitable only for wet season activity; or they are active year round by virtue of desiccation-resistant leaves or green stems with considerable water storage capacity [7]. However, Farrant et al. [13] comment that desiccation tolerance in ferns has been poorly studied.

ABA, H_2_O_2_, and SA can be used to induce plant defense responses against drought stress [14, 15]. The ABA level increases as a result of drought stress and plays an important role in regulating the responses of plants to drought stress [16]. The CAM pathway is one of the most important adaptations of some epiphytic ferns to water deficit. In the facultative halophyte* Mesembryanthemum crystallinum* L. (Aizoaceae), ABA and water stress induce the facultative CAM pathway [17].

This work aimed to provide more insight into the responses of the epiphytic fern* E*.* luridum* to water stress. To accomplish this, anatomical and physiological studies were conducted. Specifically, three treatments were analyzed: control, water deficit, and ABA. Physiological studies were conducted through analysis of RWC, photosynthetic pigments, RLC, photosynthetic parameters, and malate content after exposure to water stress and ABA treatment in a CAM context.

## 2. Material and Methods

### 2.1. Plant Collection and Acclimation

Plants of* E. luridum* were collected from the natural habitat in the Environmental Conservation Unit “Desterro” (UCAD), located in northwestern Florianópolis, Santa Catarina, Brazil (27°31′50′′S 0.8, 48°30′44′′W 0.3), an area of 495 hectares comprising about 1% of the total area of the island. The unit is managed by the Federal University of Santa Catarina (UFSC). Plants were transported to the greenhouse of the Department of Botany where they were acclimated for six months. The plants were tied and placed in brackets on the walls. During the test period, the temperature of the greenhouse varied from 20 to 30°C during the day and around 15°C at night. The relative humidity was monitored and ranged between 45 and 83%. The photosynthetic photon flux density (PPFD) inside the greenhouse alternated between 43 and 85 *μ*mol m^−^² s^−1^. Irradiance was measured at the middle region of the fronds with LI-190 sensor (LI-COR Instruments, USA) connected to the LI-250 light meter (LI-COR Instruments, USA), which was placed close to plants. The plants were watered daily with distilled water to maintain high humidity. Once a week, plants were irrigated with Hoagland's solution (20%) (v/v) [18]. After the acclimation period, the plants were divided into three groups, each subjected to different treatments: (1) control plants irrigated daily for two weeks, (2) plants subjected to drought stress for a period of one week (without irrigation), and (3) plants irrigated 5 times a week with a solution of 10 *μ*M ABA for a period of fifteen days. Treatments were made in triplicate.

### 2.2. Anatomical Analysis of Fronds

For* in vivo* structural analysis of the fronds, we performed longitudinal and paradermic cross-sections, both done freehand with a razor blade using polystyrene as support material. These sections were placed on a slide with water and covered with a coverslip. The stomata count was performed in triplicate in the median region of the abaxial surface with the aid of an optical microscope Leica DM2500 (Leica, Wetzlar, Germany), and images were captured with an attached digital camera (Leica DFC295, Leica, Wetzlar, Germany).

Structural analysis of the fronds was also performed on samples fixed in 2.5% glutaraldehyde in sodium phosphate buffer 0.1 M, pH 7.2, washed in the same buffer, dehydrated in an ethanol series, and preserved in ethanol 70° GL [19]. Some samples were infiltrated in a mixture of polyethylene glycol (PEG) 1500 and 70% ethanol (1 : 1) for 24 hours in an oven at 60°C. They were incubated again in an oven for 24 hours in pure PEG 1500. Following this incubation, the samples were embedded in PEG 1500. Other samples were infiltrated in hydroxyethyl methacrylate (Jung's Historesin, Leica, Wetzlar, Germany). The samples preserved in ethanol 70° GL were dehydrated in an ethanol series up to 96° GL and processed according to the manufacturer's instructions. The samples embedded in PEG and Historesin were sectioned by rotary microtome (Leica RM 2125 RT, Leica, Wetzlar, Germany) and stained with safranin [20] or Sudan IV for lipids [21]. Twenty measurements of cuticle thickness were performed. The material was examined under an optical microscope (Leica DM2500, Leica, Wetzlar, Germany), and images were captured with an attached digital camera (Leica DFC295, Leica, Wetzlar, Germany).

For scanning electron microscopy analysis, frond samples were subjected to total dehydration in ethanol series and kept in ethyl ether for 48 h at 20°C. Afterwards, the sample containers were opened and kept in laminar flow to favor the complete evaporation of the ether. The samples were then placed on aluminum brackets with the aid of double-sided carbon tape. Frond samples were metalized with 30 nm of gold in a Bal-Tec CED 030 metalizer (Bal-Tec AG, Balzers, Liechtenstein). A scanning electron microscope (SEM) (JEOL JSM-6390LV, JEOL, Tokyo, Japan) was used to analyze the samples.

All anatomical analyses were performed with median region of control plants fronds, disregarding ribs.

### 2.3. Determination of RWC and Malate Contents

After each treatment, 1.0 g fresh mass (FM) of frond discs for each replicate (*n* = 3) was removed and placed in flasks containing distilled water for 180 min under a PPFD of 20 *μ*mol m^−2^ s^−1^ at 25 ± 2°C to obtain the turgid mass (TM). Frond segments were then dried in an oven at 60°C for 24 h and subsequently weighed to obtain the dry mass (DM). The RWC values were calculated in fully expanded mature fronds and were expressed by the equation RWC = [(FM − DM)/(TM − DM)]∗100 [22].

Malate contents were evaluated according to Möllering [23] in order to verify differences in malate content between night and day (mM): (ΔMalate = (malate)  night − (malate)  day). Three samples (1.0 g each) were taken from fresh frond material at 6:00 h and at 18:00 h from each treatment. They were immediately immersed in liquid nitrogen (−192°C) with the aim of conserving and stopping all enzymatic reactions until analysis. For the extraction, samples were homogenized in 10 mL of distilled water at 98°C, kept in water bath at the 98°C for 10 min, and subsequently centrifuged at 3.500 g for 10 min. The precipitate was discarded and the extracts were kept at room temperature. Aliquots of 100 *μ*L were used to quantify the malate through the K-LMALL enzymatic kit (Megazyme International Ireland Limited, Ireland). Analyses were performed in a spectrophotometer (Biospectro, SP-220, EQUIPAR Ltda., Curitiba, Brazil) at 340 nm and in triplicate. Malate content was expressed in *μ*mol g^−1^ DM.

### 2.4. Analysis of Photosynthetic Pigment Contents and Photosynthetic Parameters

The photosynthetic pigments were analyzed according to Lichtenthaler [24] using samples of 1.0 g of fresh material (*n* = 3). For extraction, frond samples were taken from liquid nitrogen and immediately homogenized in 10 mL of aqueous acetone (80% v/v) for 5 min, using a mortar. The homogenate was centrifuged for 10 min at 3.500 g, and the volume was adjusted to 10 mL. The absorbances at 470 nm, 646 nm, and 663 nm were analyzed with a UV-Vis spectrophotometer (SP-220, Biospectro, EQUIPAR Ltda., Curitiba, Brazil).

The emission of chlorophyll a fluorescence of* E*.* luridum* frond was evaluated using a pulse amplitude-modulated fluorometer (Diving-PAM, Underwater Fluorometer, Walz, Effeltrich, Germany), equipped with an optical fiber 5.5 mm in diameter and a blue diode (470 nm) as the light source. During the testing period, the recorded temperature was 25 ± 2°C. The analyses were carried out between 10:00 and 12:00 h. For each of the three treatments, including control, water deficit, and application of 10 *μ*M ABA, plants were previously acclimated to the dark for 25 min before the application of the first pulse of saturating light. The experiments were performed in triplicate (*n* = 3). Transient chlorophyll a fluorescence was not measured. Ten rapid curves were obtained for each replicate (thirty photosynthesis curves for each treatment). The minimum fluorescence (*F*_*o*_) was obtained by exposing samples to a measured modulated light (ML) (0.1 *μ*mol photons m^−2^ s^−1^), and the maximum fluorescence (*F*_*m*_) in the dark-adapted samples was obtained by applying a saturating light (SL) pulse (9.000 *μ*mol m^−2^ s^−1^). Using the RLC option, the light curves were obtained by applying a series of eight pulses of saturating light (SL), each followed by exposure to crescent actinic light (AL) (2–2250 *μ*mol m^−2^ s^−1^ PPFD). The parameters of pulse-amplitude modulation (PAM), that is, electron transport rate (ETR), were calculated using the WinControl software [25]. During the measurement of RLC, each SL after an AL period produced a light-adapted maximum fluorescence (*F*_*m*_′) and light acclimated steady-state fluorescence (*F*′). The ETR between photosystems II and I (PSII and PSI) was estimated using the following equation: ETR = ΦPSII × PPFD × 0.5 × 0.84, where ΦPSII is the effective photochemical quenching yield of PSII obtained by (*F*_*m*_′ − *F*′)/*F*_*m*_′, PPFD is the photosynthetic photon flux density during exposure to actinic light, 0.5 is related to the incident photon between PSII and PSI, and 0.84 is the fraction of incident quanta absorbed by the frond [26–30].

All analyses were performed with median region of fronds, disregarding ribs.

### 2.5. Statistical Analysis

Data were analyzed by Excel and BioEstat software. Data were expressed as mean ± standard deviation. The analysis of variance (multifactor ANOVA) was followed by the mean comparison test (Tukey 5%) for data with normal distribution and homoscedasticity [31]. To quantitatively compare RLCs using parametric statistics, some descriptive parameters were used, namely, maximum electron transport rate (ETR_max⁡_) and *I*_opt_ (optimal irradiance). ETR data were plotted as rapid light curves (RLC = *P* versus *I*, where *P* is the photosynthesis in ETR and *I* is the irradiation pulses applied to draft the curves). Both parameters were plotted in a waiting-in-line equation (*y* = *x* · *e* ^−*x*^) using an Excel macro (Microsoft Office Excel 2010) [32]. The empirical model for *P* (ETR) versus *I* (irradiance) was first used by Gloag et al. [33], who applied a suitable mathematical model for photosynthesis (*P* = *A* · *k*_*w*_ · *I* · *e* ^−*k*_*w*_·*I*^). This equation calculated the ETR_max⁡_. The ETR_max⁡_ occurs at an irradiance value of 1/*k*_*w*_, the *I*_opt_. The adjusted values for constants (*A*) and (*k*_*w*_) were determined using the equation described by Gloag et al. [33].

## 3. Results 

### 3.1. Anatomical Analysis of Frond

**Figure 1:**
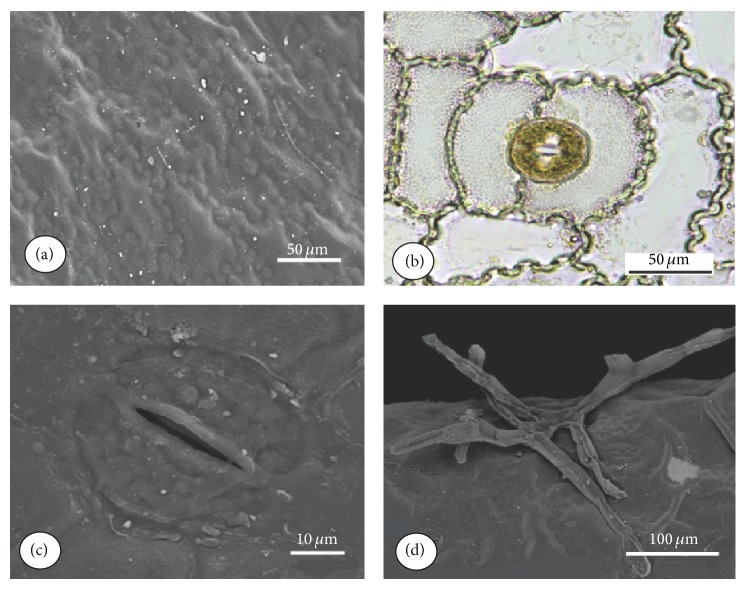
Details of SEM and LM images of* Elaphoglossum luridum* epidermis. (a) SEM image of adaxial epidermis cells. (b) LM image of polocytic stomata and epidermal abaxial cells with sinuous anticlinal walls. (c) SEM image of the polocytic stomata on the abaxial surface. (d) SEM image of branched trichome on the abaxial epidermis.

**Figure 2:**
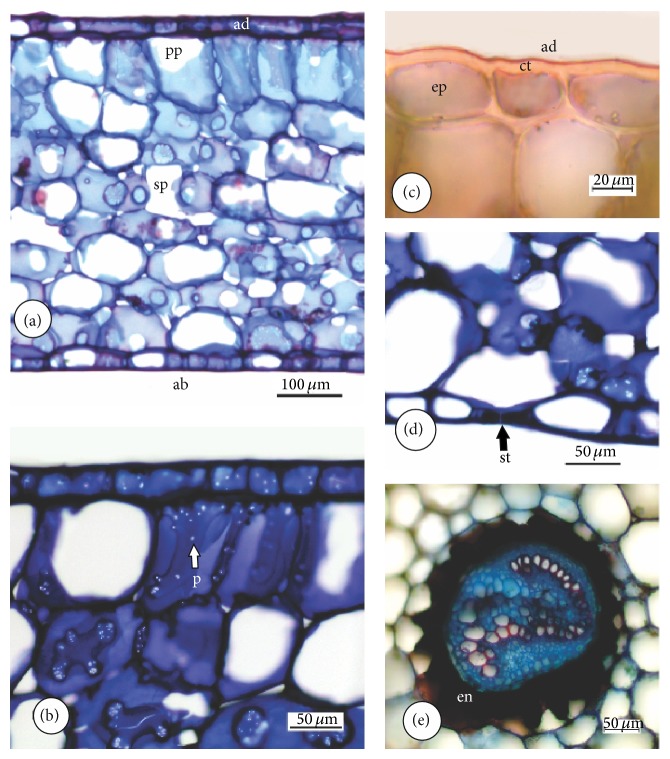
Cross-sections of* Elaphoglossum luridum* frond in LM. (a) Mesophyll consists of parenchyma tending to palisade (pp), spongy parenchyma (sp), and uniseriate epidermis, on both sides of the frond. (b) Details of the abaxial tissues showing pits in the spongy mesophyll cell walls. (c) Details of the cuticle showing a light positive Sudan IV reaction. (d) Details of stomata on the abaxial surface. (e) Details of the vascular tissue. Ab: abaxial epidermis, ad: adaxial epidermis, ct: cuticle, en: endodermis, ep: epidermis, p: pit, pp: palisade parenchyma, sp: spongy parenchyma, and st: stomata.

The epidermal cells of the fronds of* E*.* luridum* have various shapes, tending to elongation in the longitudinal direction of the frond, and the anticlinal cell walls are sinuous (Figure 1(a)). The stomata are of the polocytic type, in which the guard cells have a subsidiary cell in a horseshoe shape (Figures 1(b) and 1(c)). Multicellular trichomes, like scales with long branches (Figure 1(d)), occur on both sides of the frond, but in higher numbers on the abaxial surface. The frond blade has an average thickness of 0.54 mm (Figure 2(a)). The mesophyll (Figure 2(a)) consists of parenchyma tending to palisade and spongy parenchyma presenting cells with large vacuoles; the spongy mesophyll fills the remaining part, presenting rounded chlorophyllous cells and large intercellular spaces ranging from ten to eleven layers of cells presenting a large number of pits (Figure 2(b)). The cuticle is relatively thick (2.1 ± 0.27 *μ*m), showing a light positive Sudan IV reaction to cutin in the peripheral layer (Figure 2(c)). The frond is of the hypostomatic type, with stomata restricted to the abaxial epidermis, randomly oriented, showing a density of 33 stomata per mm^2^ (Figure 2(d)). The meristele (Figure 2(e)) is bounded externally by pericycle, with one or two cell layers containing a concentric vascular structure of the anficrival type. The xylem, positioned internally to phloem, has a V-shape.

### 3.2. Determination of RWC and Malate Contents

**Figure 3:**
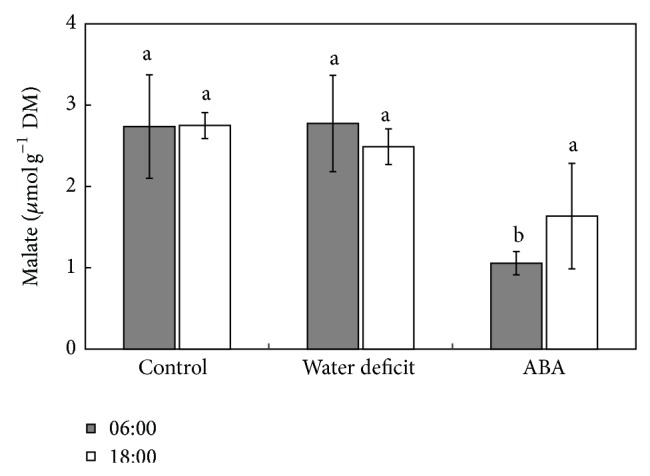
Daily fluctuation (6:00h–18:00 h) in malate content of fronds of* Elaphoglossum luridum* under different treatments. The data are presented as mean ± SD. Lowercase letters indicate the groups differentiated by ANOVA followed by Tukey's test; *P* > 0.05 (*n* = 3).

No statistically significant difference was observed in RWC in the* E*.* luridum* frond between control plants and plants under water deficit or ABA treatments. However, an increase of 3.28% RWC was seen in plants irrigated with ABA compared to plants subjected to water deficit (Table 1). No fluctuations were observed in malate contents between night and day in control plants and plants subjected to water stress. However, a decrease in total malate contents was observed at 6:00 h in plants treated with ABA for 15 days compared to control plants and plants subjected to water stress (Figure 3).

### 3.3. Analysis of Photosynthetic Pigment Contents and Photosynthetic Parameters

**Figure 4:**
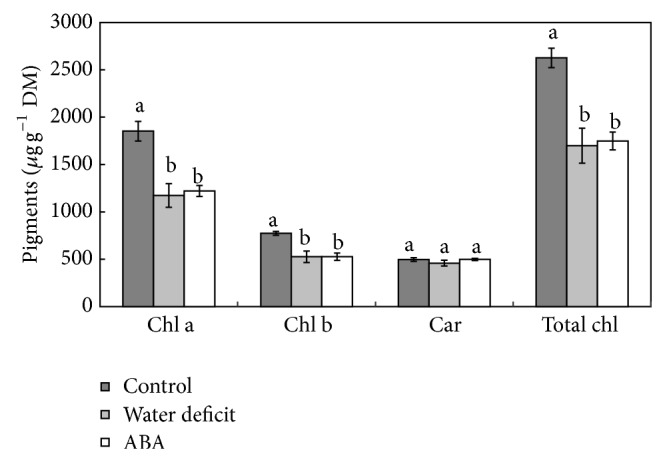
Chlorophyll (Chl a, Chl b, and total Chl) and carotenoid (Car) contents in fronds of* Elaphoglossum luridum* under different treatments. The data are presented as mean ± SD. Lowercase letters indicate the groups differentiated by ANOVA followed by Tukey's test; *P* > 0.05 (*n* = 3).

**Figure 5:**
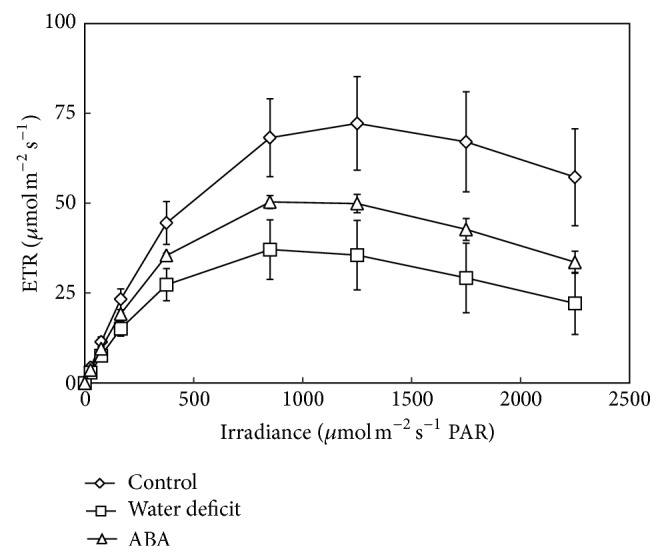
Rapid light curve (RLC) plotted as ETR (*P*) versus irradiance (*I*) in fronds of* Elaphoglossum luridum* under different treatments. The data are presented as mean ± SD. Lowercase letters indicate the groups differentiated by ANOVA followed by Tukey's test; *P* > 0.05 (*n* = 3).

In the* E*.* luridum* frond, results of this study show that the contents of total chlorophyll, as well as chlorophyll a and b, were significantly lower under water stress treatment for seven days and under ABA treatment for fifteen days compared to the control (Figure 4). The analysis of chlorophyll a fluorescence in* E*.* luridum* (Table 2) showed significant differences among the photosynthetic parameters analyzed, *I*_opt_, ETR_max⁡_, and the alpha *α* (photosynthetic efficiency), under water stress for seven days and ABA irrigation for fifteen days compared to control plants. These results can also be observed in rapid light curves (Figure 5) where the values of *I*_opt_, ETR_max⁡_, and *α* declined during stress treatments. Water stress caused a 26.8% reduction in *I*_opt_ and after ABA application a reduction of 16.9%. ETR_max⁡_ was also observed under water stress (50.5%) and ABA treatment (32.5%). For *α*, the reduction was 36.1% for water stress and 20.4% for ABA treatment.

## 4. Discussion

*E*.* luridum* presents coriaceus and succulent fronds, showing a parenchyma tending to the palisade and chlorophyllous spongy parenchyma which presents large vacuoles to store water. The relatively thick cuticle in the outer periclinal walls of epidermal cells is an aspect that should contribute to the maintenance of water balance. In general, cuticle thickness varied from 0.1 to 10 *μ*m, considering leaves, fruits, and other primary organs of higher plants [34, 35]. According to Hietz and Briones [8], these features are commonly found in epiphytic ferns and are part of a list of attributes responsible for maintaining water during recurring periods of drought. Sinuous anticlinal cell walls were observed in the epidermis of* E. luridum* frond. Krauss [36] commented that the tendency toward sinuosity represents mechanical adaptations to avoid collapse during expansion and contraction of the leaf by the entrance and exit of water.* E. luridum* shows branched trichomes in the epidermis of the foliar blade. Scales with similar anatomical structure were also found in* Pleopeltis mexicana* (Fée) fronds and* Elaphoglossum petiolatum* Bonap., which probably increase the capacity of fronds to absorb water without increasing cuticle water loss [8]. The scales have evolved independently in ferns and bromeliads, but they are treated as an important structure for water absorption in epiphytes [8].

Plants of* E*.* luridum* were able to maintain RWC around 90% after water stress. This species is homoiohydrous. Tausz et al. [37] observed similar results for epiphytic ferns, including* Elaphoglossum glaucum* T. Moore and* E*.* petiolatum* Bonap., which are able to maintain high contents of RWC under short-term stress. Hietz and Briones [8] demonstrated that the leathery fronds of* E. glaucum* showed a lower rate of cuticle transpiration among the studied species. In* E. luridum* frond, the exogenous application of ABA for a period of fifteen days slightly increased RWC in contrast to plants subjected to drought stress. In fact, ABA does play a role in the control of water balance in ferns. When fronds of* Polypodium virginianum* L., a desiccation-tolerant fern, were incubated in ABA for 24 h prior to silica-drying, the amount of water lost was reduced, resulting in survival of the fronds upon subsequent rehydration [38]. Ruszala et al. [39] found that stomatal responses of the lycophyte* Selaginella uncinata* to ABA and CO_2_ are directly comparable to those of the flowering plant* Arabidopsis thaliana*.

After water stress and ABA treatment, malate content found in* E. luridum* seems to be many times lower than the content found in plants having the CAM pathway, suggesting the absence of CAM pathway in* E*.* luridum* frond based on day/night variation. In* M*.* crystallinum* L. (Aizoaceae), malate contents were higher than 75 *μ*mol·g^−1^ FM after ABA treatment [17]. In pineapple, Δ malate reached almost 500 *μ*mol·g^−1^ DM after 25 days of water stress [40]. On the other hand, malate content decreased in fronds of* E*.* luridum* after ABA treatment, seeming to downregulate malate metabolism. The ABA mode of action is linked to diurnal stomatal movements; the elevated level of ABA biosynthesis in the dark phase of the day is responsible for stomatal closure. In the evening, ABA biosynthesis outweighs ABA catabolism in the guard cells, leading to stomatal closure. Under drought stress conditions, ABA could reach a concentration high enough to cause ion efflux and inhibition of sugar uptake by the guard cells. ABA can also stimulate gluconeogenic conversion of malate into starch, thus reducing stomata apertures [41]. Based on these lines of evidence, we suggest that ABA could reduce malate contents in fronds of* E*.* luridum*, not only in guard cells, but also in the mesophyll cells, especially at the night.

In this paper, we observed a small decrease in chlorophyll contents, but not in carotenoid contents, after water stress and ABA treatment in* E*.* luridum* frond. Tausz et al. [37], working with photosynthetic pigments in different species of epiphytic ferns in a Mexican rainforest, noted a response similar to that obtained in this work; that is, that total chlorophyll content decreased under water stress induced in* Polypodium plebeium* Schltdl. and Cham.,* E*.* petiolatum*,* Phlebodium areolatum*, and* Asplenium cuspidatum* Lam. The reduction in chlorophyll content can be attributed to the high degradation rate of these pigments, which is higher than the biosynthesis under drought stress conditions [42]. On the other hand, chlorophyll was retained in* Mohria caffrorum* (L.) Dev. (Anemiaceae) during drying in both DT and DS forms of the plant [13].

Drought stress is potentially harmful by enhancing the production of reactive oxygen species (ROS) [43]. Drought stress is known to inhibit photosynthetic activity in tissues. Downregulation of PSII activity results in an imbalance between the generation and utilization of electrons [44]. In this study, water deficit and application of ABA decreased ETR_max⁡_ and *I*_opt_ and seemed to negatively regulate the rate of electron transport in fronds of* E*.* luridum*. Similarly, in* M*.* caffrorum*, the quantum efficiency of photosystem II (FV/FM) of DT leaves declined once the water level dropped below 70% RWC, but it recovered to predesiccation levels upon rehydration, suggesting that little damage had occurred in the photosynthetic apparatus in those tissues, or, in the alternative, that rapid repair had occurred [13]. According to Maxwell and Johnson [45], the drop of RLC under stress indicates photoinhibition of PSII. Therefore, our data suggest that both water stress and ABA moderately inhibited PSII activity in frond.

Our results show that* E*.* luridum* frond is coriaceus and succulent, presenting trichomes, relatively thick cuticle, and sinuous cell walls in both abaxial and adaxial epidermis. Since it is homoiohydrous, it is able to maintain RWC under mild water stress and after application of ABA, but, at the same time, these factors reduced chlorophyll contents and ETR. This species did not show the ability to regulate the C_3_ metabolism required for CAM under water stress and ABA, but it did show morphological adaptations strong enough to withstand periods of drought.* E. luridum* responded to ABA, maintaining higher levels of RWC and lower contents of nocturnal malate.

## Figures and Tables

**Table 1:** Changes in the RWC, in fronds of *Elaphoglossum luridum* under different treatments: control, water deficit (seven days), and ABA-treated fronds (fifteen days) (10 *μ*M). The data are presented as mean ± SD. *Lowercase letters* indicate the treatments differentiated by ANOVA followed by Tukey's test; *P* > 0.05 (*n* = 3).

Treatment	RWC (%)
Control	89.37 ± 0.60^ab^
Water deficit	87.22 ± 1.36^b^
ABA	90.50 ± 0.56^a^

**Table 2:** Rapid light curve (RLC) parameters plotted as ETR (*P*) versus irradiance (*I*) in sporophytes of *Elaphoglossum luridum* under different treatments. The data are presented as mean ± SD. *Lowercase letters* indicate the treatments differentiated by ANOVA (between columns) followed by Tukey's test; *P* > 0.05 (*n* sample/curves = 3/30). *A*: scaling constant for the height of the light curve; ETR_max_: maximum electron transport rate; *I*_opt_: optimal irradiance; and *K*_*w*_: scaling constant for the *x*-axis of the light curve.

	Control	Water deficit	ABA stress
*I*_opt_ (*μ*mol photon m^−2^ s^−1^)	1.208 ± 81.0^a^	884.0 ± 67.0^b^	1.004 ± 33.0^b^
ETR_max_ (*μ*mol electron m^−2^ s^−1^)	76.0 ± 9.0^a^	37.0 ± 8.0^b^	51.0 ± 2.0^b^
Alpha (*α*)	0.172 ± 0.008^a^	0.110 ± 0.01^c^	0.137 ± 0.001^b^
(*A*)	196.0 ± 35.0	102.0 ± 24.0	139.0 ± 6.0
(*K*_*w*_)	0.0008 ± 0.0001	0.0011 ± 0.0001	0.0001 ± 0.00004
Correlation coefficient *r*	0.978	0.956	0.966
*n* sample/curves	3/30	3/30	3/30

## Abbreviations

A: Scaling constant for the height of the light curve
ABA: Abscisic acid
AL: Actinic light
CAM: Crassulacean acid metabolism
DM: Dry mass
DS: Desiccation-sensitive
DT: Desiccation-tolerant
ETR: Electron transport rate
${\text{ETR}}_{\max}$: Maximum electron transport rate
FM: Fresh mass
$F_{o}$: Minimum fluorescence
$F_{m}$: Maximum fluorescence
$F_{v}$: Variable fluorescence
$F_{m}^{\prime }$: Light adapted maximum fluorescence
$F^{\prime }$: Light acclimated steady-state fluorescence
FLM: Fluorescence microscopy
$F_{v}$/$F_{m}$: Maximum photochemical efficiency of PSII
I: Irradiance
$I_{\text{opt}}$: Optimal irradiance
$K_{w}$: Scaling constant for the x-axis of the light curve
LM: Light microscopy
ML: Modulated light
PAM: Pulse-amplitude modulation
P: Photosynthesis
PAR: Photosynthetically active radiation
PEG: Polyethylene glycol
PPFD: Photosynthetic photon flux density
PS: Photosystem
RCL: Rapid light curve
RWC: Relative water content
SA: Salicylic acid
SEM: Scanning electron microscopy
SL: Saturating light pulses
ΦPSII: Actual photochemical efficiency of PSII
TCA: Tricarboxylic acid cycle
TM: Turgid mass.
